# Epidermal growth factor receptor (EGFR) biology and Wnt developmental signaling highlight research endeavors of SCBA award winners

**DOI:** 10.1186/2045-3701-2-12

**Published:** 2012-04-20

**Authors:** Kuan-Teh Jeang

**Affiliations:** 1 National Institute of Allergy and Infectious Diseases, Bethesda, MD, 20892, USA

## Abstract

This months *Cell and Bioscience* highlights review articles by Mien-Chie Hung on EGFR biology and Yingzi Yang on Wnt signaling. Dr. Hung was the 2011 Society of Chinese Bioscientists in America (SCBA) Presidential Award winner. Dr. Yang was the 2011 SCBA Outstanding Young Investigator Award winner.

## Commentary

At the 2011 biennial international meeting held in Guangzhou, China, the Society of Chinese Bioscientists in America (SCBA) recognized two of our finest members for their scientific accomplishments and future potential in biomedical research. SCBA awarded its Presidential Award to Dr. Mien-Chie Hung (MD Anderson Cancer Center, Houston, USA) and its Outstanding Young Investigator Award to Dr. Yingzi Yang (National Institutes of Health, Bethesda, USA). I have previously written on the importance of prizes/awards for encouraging and rewarding research endeavors [1]. In this months *Cell and Bioscience*, we have the privilege of publishing two review articles from Hung [2] and Yang [3] reprising their contributions to cancer and developmental biology and posing future directions for their respective scientific fields.

Professor Mien-Chie Hung is an internationally renowned cancer biologist. He graduated from the National Taiwan University with his bachelors degree in 1973, and received his Ph.D. degree from Brandeis University in 1983. After training with Robert Weinberg at MIT, he joined the faculty of the University of Texas MD Anderson Cancer Center in 1986 as an Assistant Professor in the Department of Tumor Biology. Professor Hung is currently the Chairman of the Department of Molecular and Cellular Oncology, and the Vice President for Basic Research at the University of Texas MD Anderson Cancer Center, Houston, Texas.

Amongst his many honors, Professor Hung is an Academician of Academia Sinica, Taiwan.

Hung is a pioneering leader in our understanding of the normal biology and abnormal cancer transforming functions of epidermal growth factor receptor (EGFR) family members. He is well-recognized to have made the unexpected, but seminal, discovery that receptor tyrosine kinases like EGFR and HER2/neu can be found in the nucleus, can bind to specific DNA sequences, and can exert nuclear transcriptional activity. In his article [2] in this months *Cell and Bioscience*, Hung reviews the mechanisms that traffic EGFR, normally a cell surface membrane receptor, into the nucleus and into a variety of cellular organelles such as the Golgi, the endoplasmic reticulum, and the mitochondria.

Dr. Yingzi Yang was a Ph.D. student with L. Niswander at the Sloan Kettering Cancer Institute and a postdoctoral fellow with A. McMahon at Harvard University. As a graduate student, Dr. Yang discovered that vertebrate limb development is coordinately regulated in three dimensions. As a postdoctoral fellow, she focused on Wnt and Hedgehog (Hh) signaling in limb development using mouse genetics. She was one of the first to postulate that Sonic hedgehog (Shh) patterns the anterior-posterior axis of the developing limb by forming a morphogen gradient. In August 2000, Dr. Yang was recruited to the National Institutes of Health, Bethesda, Maryland, where she is now a tenured senior investigator in the National Human Genome Research Institute. Last year, Dr. Yang was also recognized by the NIH Asian and Pacific American Organization (APAO) with its annual outstanding Asian American scientist award.

Yangs current focus is on cell-to-cell signaling in limb development and its impact on skeletal morphogenesis. She also studies how Wnt signaling affects mechanisms of planar cell polarity (PCP). Her recent work has shown that PCP regulation is critical to the development of left-right symmetry. In her article in this months *Cell and Bioscience*, Yang reviews how Wnt proteins transduce their signals through several distinct pathways and regulate vertebral development via quantitative and directional information. She raises the importance of these insights toward our understanding of diseases such as Robinow syndrome, brachydactyly type B1, and spina bifida.

SCBA is proud of outstanding members like Mien-Chie Hung and Yingzi Yang. We hope that you enjoy reading and learning from their review articles.
